# Targeting of the *Plzf* Gene in the Rat by Transcription Activator-Like Effector Nuclease Results in Caudal Regression Syndrome in Spontaneously Hypertensive Rats

**DOI:** 10.1371/journal.pone.0164206

**Published:** 2016-10-11

**Authors:** František Liška, Renata Peterková, Miroslav Peterka, Vladimír Landa, Václav Zídek, Petr Mlejnek, Jan Šilhavý, Miroslava Šimáková, Vladimír Křen, Colby G. Starker, Daniel F. Voytas, Zsuzsanna Izsvák, Michal Pravenec

**Affiliations:** 1 Institute of Biology and Medical Genetics, 1st Medical Faculty, Charles University in Prague, Prague, Czech Republic; 2 Institute of Experimental Medicine, Czech Academy of Sciences, Prague, Czech Republic; 3 Institute of Physiology, Czech Academy of Sciences, Prague, Czech Republic; 4 University of Minnesota, Minneapolis, Minnesota, United States of America; 5 Max Delbrück Center for Molecular Medicine, Berlin, Germany; Imperial College London, UNITED KINGDOM

## Abstract

Recently, it has been found that spontaneous mutation *Lx* (polydactyly-luxate syndrome) in the rat is determined by deletion of a conserved intronic sequence of the *Plzf* (Promyelocytic leukemia zinc finger protein) gene. In addition, *Plzf* is a prominent candidate gene for quantitative trait loci (QTLs) associated with cardiac hypertrophy and fibrosis in the spontaneously hypertensive rat (SHR). In the current study, we tested the effects of *Plzf* gene targeting in the SHR using TALENs (transcription activator-like effector nucleases). SHR ova were microinjected with constructs pTAL438/439 coding for a sequence-specific endonuclease that binds to target sequence in the first coding exon of the *Plzf* gene. Out of 43 animals born after microinjection, we detected a single male founder. Sequence analysis revealed a deletion of G that resulted in frame shift mutation starting in codon 31 and causing a premature stop codon at position of amino acid 58. The *Plzf*^*tm1Ipcv*^ allele is semi-lethal since approximately 95% of newborn homozygous animals died perinatally. All homozygous animals exhibited manifestations of a caudal regression syndrome including tail anomalies and serious size reduction and deformities of long bones, and oligo- or polydactyly on the hindlimbs. The heterozygous animals only exhibited the tail anomalies. Impaired development of the urinary tract was also revealed: one homozygous and one heterozygous rat exhibited a vesico-ureteric reflux with enormous dilatation of ureters and renal pelvis. In the homozygote, this was combined with a hypoplastic kidney. These results provide evidence for the important role of *Plzf* gene during development of the caudal part of a body—column vertebrae, hindlimbs and urinary system in the rat.

## Introduction

Deletions of the chromosomal region 11q23 are known in human resulting in a phenotype including mental retardation, craniofacial dysmorphism, microcephaly and short stature. Within the chromosomal region 11q23, the promyelocytic leukaemia zinc finger (PLZF) gene encodes a DNA sequence-specific transcriptional repressor (OMIM 176797). A similar mutation also exists in animal models. In Wistar outbred rats, spontaneous mutation *Lx* (polydactyly-luxate syndrome) was originally detected and fixed in the PD/Cub (polydactylous) inbred strain and transferred to genetic backgrounds of the BN-*Lx* (Brown Norway) and SHR-*Lx* (spontaneously hypertensive rat) congenic strains [1,2]. Based on the identification of the mouse *lu* (Green's luxoid) spontaneous mutation as a nonsense point mutation in the *Plzf* (Promyelocytic leukemia zinc finger protein) gene [3,4] and conservation of orthologous regions of mouse chromosome 9 and rat chromosome 8, that include *lu* and *Lx* genes, respectively, *Plzf* was an obvious candidate for the *Lx* mutation. Sequence and fine mapping analyses revealed deletion of a conserved intronic sequence of the *Plzf* gene to be responsible for the *Lx* phenotype [5]. PLZF is a multifunctional transcriptional repressor involved in major biological processes during development, for instance in stem cell self-renewal, including hematopoietic stem cells, neural progenitor cells or spermatogonial progenitor cells and in stem cell differentiation, including myeloid differentiation, limb bud development, osteogenesis, chondrogenesis or lymphoid differentiation [6].

Recently, linkage analyses in BXH/HXB recombinant inbred (RI) strains, derived from SHR and BN-*Lx* progenitors, and in SHR-*Lx* minimal congenic strain, identified *Plzf* as a candidate gene predisposing the SHR to left ventricular hypertrophy and cardiac interstitial fibrosis [7,8]. In addition, it was shown that *Plzf*^*-/-*^ mice are protected against cardiac hypertrophy induced by angiotensin II [9]. In the current study, we used TALEN technology to target the *Plzf* gene in the SHR to study the effect of *Plzf* knockout on cardiac hypertrophy and fibrosis. Surprisingly, we found that, contrary to the mouse, over 95% of *Plzf*^*-/-*^ homozygotes die shortly after birth. All homozygous animals exhibited manifestations of a caudal regression syndrome including tail anomalies and serious reduction deformities of long bones, and oligo- or polydactyly on the hindlimbs. Impaired development of the urinary tract was also found. The heterozygous animals only exhibited the tail anomalies.

## Results

### Derivation of SHR rats with targeted *Plzf* gene

**Fig 1:**
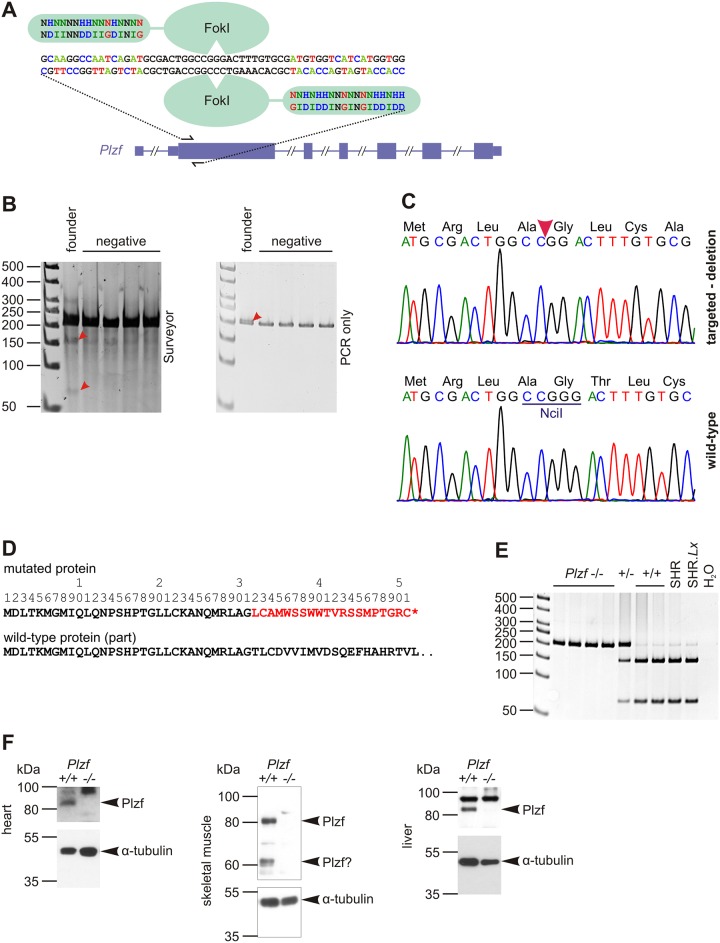
Derivation of *Plzf*-null rat. **(A)** Design of the TAL effector endonuclease to target exon 2 (first coding exon) of *Plzf* gene. Two key aminoacid residues recognising each base of the target are shown. FokI = FokI endonuclease domain. **(B)** Screening of pups from microinjected zygotes by Surveyor endonuclease—left panel, second lane (first sample) represents the founder with cleaved heteroduplex (arrowheads). Interestingly, the heteroduplex was visible on native polyacrylamide elecrophoresis gel (without Surveyor treatment)–right panel, arrowhead. **(C)** Sequencing of the founder´s *Plzf*–comparison of the mutated and wild-type allele. Note NciI recognition site in the wild-type sequence. **(D)** Comparison of predicted amino acid sequence of the wild-type and targeted *Plzf* shows truncation of the protein. **(E)** Example of genotyping the targeted mutants with PCR followed by restriction digestion by NciI. Mutant allele is not cleaved. **(F)** Western blotting using N-terminal anti-PLZF antibody in the heart, liver, kidney and skeletal muscle in *Plzf*^*-/-*^ mutant homozygote and wild-type animals.

Out of 43 animals born after microinjection, we detected a single male founder (Fig 1B). Sequence analysis revealed a deletion of G at position 91 of the coding sequence (c.91delG, Fig 1C) that resulted in a frameshift at glycine 31 (p.Gly31fs). The frameshift caused incorporation of 20 aberrant amino acids downstream of the deletion followed by a stop codon (Fig 1D). We bred the founder with SHR to generate more heterozygous animals, and then intercrossed the heterozygotes to obtain knock-out homozygotes. We monitored the breeding by PCR and NciI digestion (see Methods and Fig 1E). No PLZF protein was detected in the tissues isolated from perinatal SHR-*Plzf*^*-/-*^ homozygotes (Fig 1F).

### Prenatal study

**Fig 2:**
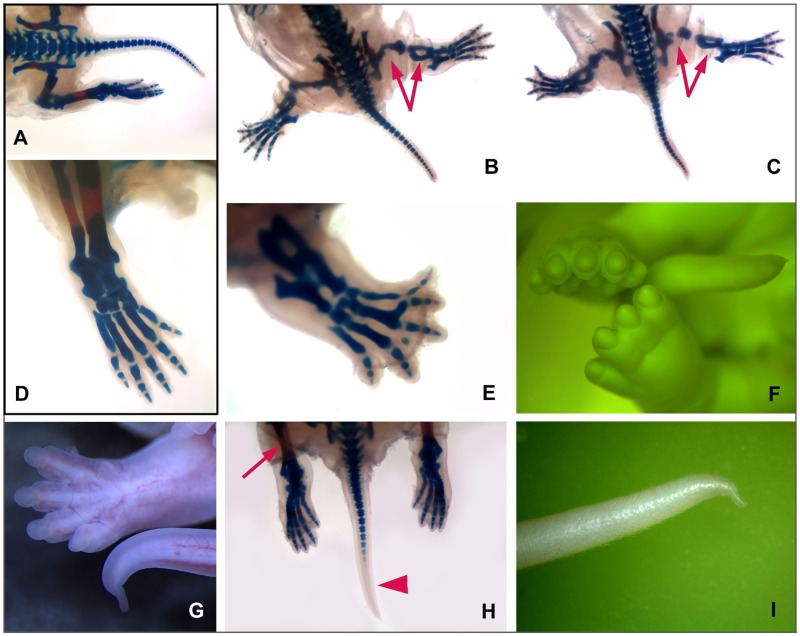
Caudal regression syndrome in fetuses of *Plzf* deficient rats. In comparison to wild-type **(A, D)**, or heterozygous **(H)** rat fetuses, note the markedly reduced length and missing (delayed) ossification of the long bones (arrows) in *Plzf*^*-/-*^ rats **(B, C, E). (B)** Left foot shows normal number of 5 metatarsal bones, but a duplication of the 2^nd^ finger. In contrast, a toe is absent on the right side of this animal. **(C)** Bilateral oligodactyly. **(E)** Absence of the 1^st^ metatarsal bone and toe, and duplication of the 3^rd^ finger. **(F)** Oligodactyly on the left foot after fixation in Bouin-Hollande fluid, which gives the green colour to the specimen. **(G)** Oligodactyly and thin and bent tail tip. **(H)** The tail of the *Plzf* ^*+/-*^ rat with strong reduction of the number of tail vertebrae; note the absence of vertebrae in the terminal part of the tail. **(I)** A thin and bent tail tip in a postnatal *Plzf*^*-/-*^ rat.

According to their genotype, all 37 fetuses were ranked into three groups: 9 *Plzf*^*-/-*^, 17 *Plzf*^*+/-*^ and 11 *Plzf*^*+/+*^ animals. The mean body weight revealed a significant (p = 0.02) growth retardation of *Plzf*^*-/-*^ fetuses (mean body weight 1716±35 mg) and *Plzf*^*+/-*^ fetuses (mean body weight 1674±37 mg), when compared to *Plzf*^*+/+*^ animals (body weight 1831±35 mg). All wild-type rats were without a visible malformation (Fig 2A and 2D). However, all *Plzf*^*-/-*^ fetuses exhibited manifestations of a caudal regression syndrome: an affection of hindlimbs and tail. A reduction in the length and missing (delayed) ossification of long bones (stylopod and zeugopod) were present in 100% of rats (Fig 2B and 2C). An autopod was malformed in all rats. Among 18 hindlimbs of 9 *Plzf*^*-/-*^ fetuses, 56% and 39% of autopodes showed oligodactyly and polydactyly, respectively, and 5% were normal (Fig 2B, 2C, 2E, 2F and 2G; compare to Fig 2D). The tail was always malformed. Before fixation, only bending of the tail tip was apparent (Fig 2G). Skeleton staining revealed a reduction in the number of tail vertebrae from the normal number 27 to 23–25 (Fig 2A–2C). In contrast, the upper limb was normal in all homozygous fetuses except one, which had a polydactyly (double thumb) on its left upper limb. There were two metacarpi articulating together with a common carpal bone. The inner and outer thumb had 3 and 2 phalanges, respectively.

Among the heterozygous fetuses, only 18% exhibited signs of the caudal regression syndrome. We recorded malformation of the tail (bending of the tip) and strong reduction of tail vertebrae from a normal number 27 to 16–17. In these animals, the distal portion of the tail was only formed by soft tissues (Fig 2H). One *Plzf*^*+/-*^ rat, which exhibited reduction of tail vertebrae, had also left renal pelvis and ureter strongly extended and full of urine. A similar developmental anomaly associated with ‘vesico-ureteric reflux’ was also revealed in the postnatal *Plzf*^*-/-*^ rat (see below).

### Postnatal study

**Fig 3:**
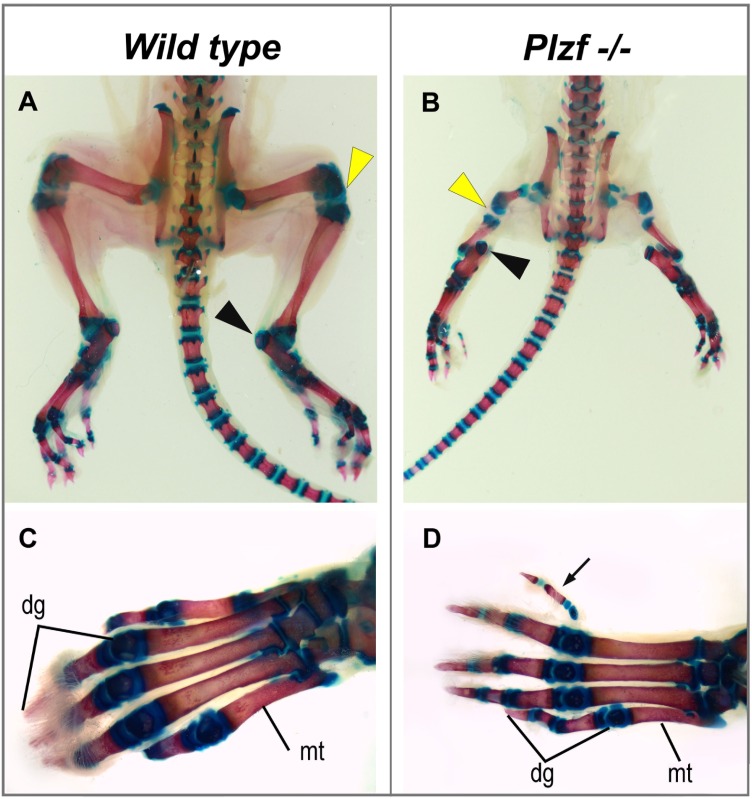
Hind-limbs in the postnatal *Plzf*^*-/-*^ and wild-type rat. **(A)** Wild-type animal has normal length of long bones, and **(C)** a foot with 5 metatarsal bones (mt) and 5 digits (dg). **(B)** The *Plzf*^*-/-*^ legs exhibit evidence of shortening of long bones, while the overall size of the foot is appropriate, but the number of metatarsal bones is reduced (absence of the 1^st^ one) while the toe itself is present **(D)**. Yellow arrow—knee, black arrow—heel.

We examined a *Plzf*^*-/-*^ rat, which survived till the age of 10 days and externally exhibited serious manifestations of the caudal regression syndrome. Its body weight was only 10.1 g, while the weight of its wild-type siblings was nearly double (18.9 g and 17.9 g, respectively). Caudal part of the body of this animal was hypoplastic and both lower extremities had markedly reduced length of the stylopod and zeugopod (Fig 3). The lower right autopod was normal but the left one missed the first metatarsal bone, while digital phalanges were reduced in size, but present (Fig 3D). The end of the tail was bent (Fig 2I) and the number of tail vertebrae reduced (from 27 to 24), similarly to what was observed in the prenatal study. The upper limbs were without a visible anomaly.

**Fig 4:**
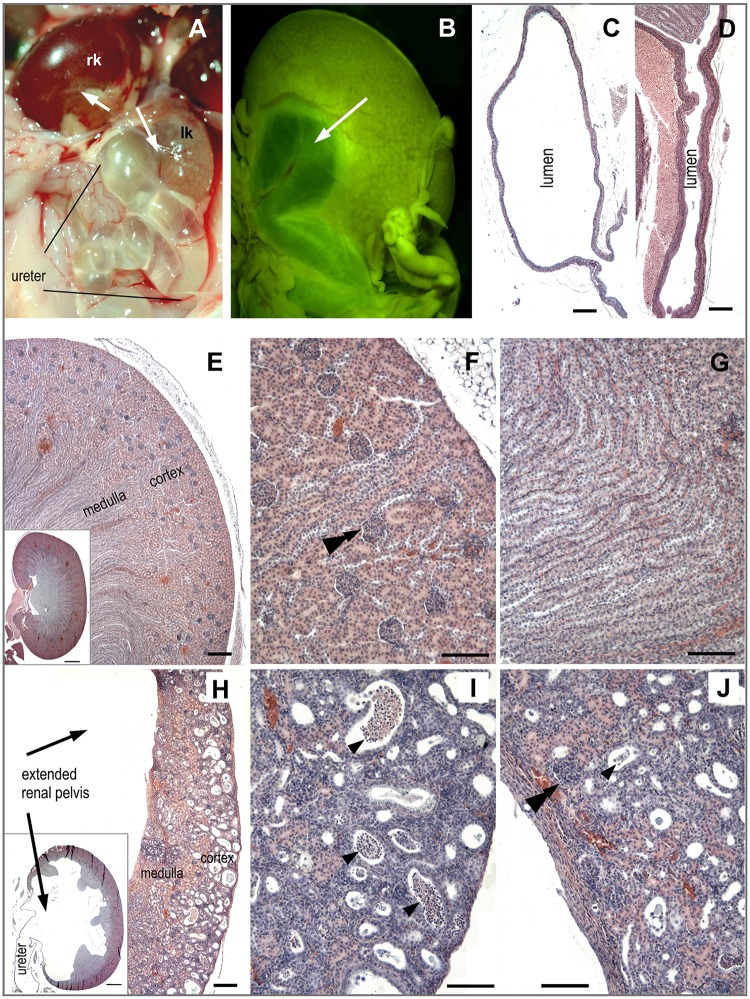
Anomaly of the urinary tract in the caudal regression syndrome in postnatal rat. **(A)** View on the kidneys and left ureter *in situ*: rk—right kidney; lk—left hypoplastic kidney with ureter enormously extended by urine (hydro-ureter). **(B)** Right kidney with extended pelvis (white arrow) after fixation in Bouin-Hollande fluid, which gives tissues a green colour. **(C, D)** Comparison between the extended ureter with a large lumen and thin wall in the mutant **(C)** and a normal urether in wild-type **(D)** rat. Histological sections of kidney in a wild-type **(E-G**) and *Plzf*^*-/-*^ **(H-J)** rat. **(E, H)** A low magnification shows basic components of kidney: cortex, medulla and renal pelvis (appearing as a free space here), which continues to ureter. Inserted box: A section shows the whole kidney, which has enormously extended pelvis and reduced renal parenchyma in the mutant rat. **(F, I)** Renal cortex; **(G, J)** medulla. Black arrow indicates an extended renal pelvis. Single arrowheads—dilated renal tubules with leucocytes. A double arrowhead—renal glomerulus. Bar—1 mm (insert in **E** and **H**); 200 μm **(C, D, E, H)**; 100 μm **(F, G, I, J)**.

The left kidney was markedly hypoplastic and much less perfused than the right one (Fig 4A). Both kidneys showed hydronephrosis, with pelvis and ureter enormously extended by urine at the expense of renal tissue (Fig 4). Compared to wild-type animal (Fig 4E–4G), the renal cortex and medulla were markedly thinner and renal tissue architecture was disrupted in the homozygous mutant pup (Fig 4H–4J). Renal glomeruli also occurred in medulla, instead of being only present in renal cortex. The renal tubules were dilated and contained a large number of leucocytes (Fig 4I and 4J).

## Discussion

In the present study, a caudal regression syndrome and significant decrease of body weight were found in the *Plzf*^*-/-*^ deficient rats. The significant decrease in body weight (growth retardation) was already found before birth, not only in *Plzf*^*-/-*^ rats, but also in the heterozygous *Plzf*^*+/-*^ animals. The growth retardation is known to be associated with inborn defects. About one-third of the variations in birth weight are determined by genetic variables in human [10].

The syndrome of caudal regression is well known in humans, being characterized by varying degrees of developmental disorders of the caudal half of the body (the lower back and limbs, caudal vertebrae). The external malformations can be associated with developmental disorders of internal organs, such as renal agenesis and dysplasia, vesicoureteral reflux (VUR) including hydronephrosis and dilated ureters, ectopic ureters, fused kidneys, absent bladder, rectovaginal and rectourethral fistulae, transposition of external genitalia, hypospadias, urethral atresia etc. [11].

All homozygous rats in the present study exhibited typical external features of the caudal regression syndrome. Only a portion of heterozygous animals presented externally a “miniform” of the syndrome (reduction of tail vertebrae). However, serious developmental disorder of the urinary tract occurred in one homozygous and one heterozygous rat: they exhibited VUR. The inherited VUR is a complex genetic developmental disorder caused by anomalous development of the urinary tract (ureter, bladder) [12,13]. The malformation of the urinary tract prevents appropriate evacuation of urine, which flows retrogradely from the bladder to the kidneys, and progressively leads to urinary tract infections, hydronephrosis, hypertension, and renal failure. The VUR and its pathological consequences are well known in human patients with caudal regression syndrome [14].

Phenotype of the *Plzf* knock-out rats is not completely consistent with the observed phenotypes of the *Plzf*^*-/-*^ and *luxoid* mouse [3,4]. Most strikingly, while the mice with null *Plzf* are viable, the rats die shortly after birth as a result of the urinary tract abnormalities. Hind limb abnormality was more pronounced compared the the *Plzf* ^*-/-*^ mice, when the frequency of missing digit I is less than 40% in the mouse [3], but 60% in the rat. Most common autopod malformation in the mouse is triphallangeal digit I [3], which was present in about 40% of the rats, however, accompanied by an extra digit preaxially. Furthermore, the rats showed severe stylopod reduction (Figs 2 and 3), which has not been described in the mouse [3]. In general, the limb phenotype of the knock-out mouse resembles better the hypomorph *Lx* mutation [5] than the more pronounced abnormality of the null rat.

In humans, a rare biallelic mutation of the PLZF gene was described. Observed malformations included bilateral absence of thumbs, aplasia or hypoplasia of ulna, delayed ossification, cleft palate, micropenis, cryptorchidism, short stature, microcephaly, and mental retardation. No renal malformations were reported [15]. This human mutation is similar to caudal regression traits observed in *Plzf*-deficient mouse and rat strains.

Based on the present data, we can conclude that the *Plzf* deficient rats are affected by the impaired development of the caudal half of the body—caudal regression syndrome, which may also include an inherited anomaly of the urinary tract. The latter anomaly causes the vesico-ureteric reflux with reflux nephropathy, which can progressively lead to renal failure and perinatal death.

## Materials and Methods

### Animals

We targeted the *Plzf* gene by TALEN method in a highly inbred strain of SHR (SHR/OlaIpcv) that has been brother x sister mated for well over 140 generations. SHR harboring KO mutation in the *Plzf* gene were derived by microinjections of fertilized ova with pTAL438/439 constructs (see next section). The adult rats were sacrified by cervical dyslocation and the rats from prenatal and perinatal studies were sacrified by decapitation. The rats were housed in an air-conditioned animal facility and allowed free access to standard diet and water. All experiments were performed in agreement with the Animal Protection Law of the Czech Republic and were approved by the Ethics Committee of the Institute of Physiology, Czech Academy of Sciences, Prague (Permit Number: 66/2014).

### The TALEN construct, sequencing, and genotyping

SHR ova were microinjected with constructs pTAL438/439 coding for homodimeric endonuclease that binds to target sequence in the first coding exon of the *Plzf* gene. pTAL438 construct was targeting sequence 5´ GCA AGG CCA ATC AGA T 3´, followed by spacer 5´ GCG ACT GGC CGG GAC TTT GTG CG 3´ and by sequence 5´ CCA CCA TGA TGA CCA CAT 3´ recognized by pTAL439 (Fig 1A). pTAL438/439 constructs were assembled as described previously [16]. We screened the pups using Surveyor (CelI) endonuclease (Transgenomics, Omaha, NE) according to published protocol [17]. We monitored cleavage of the PCR product prepared with primers “Plzf_ex2_26F”: 5´ TCC AAC TGC AGA ACC CTA GC 3´, and “Plzf_ex2_249R”: 5´ GAT CTG CTG GAA GGT TTT CG 3´ using polyacrylamide gel electrophoresis. When we obtained evidence of Surveyor cleavage, we sequenced the PCR product both directly and after cloning into pCRII-Blunt-TOPO vector (Invitrogen, Carlsbad, CA) using BigDye Terminator v1.1 Cycle Sequencing Kit and ABI PRISM 310 Genetic Analyzer (Applied Biosystems, Foster City, CA). For genotyping the offspring of the founder, we took the advantage of the induced mutation (c.91delG) that resulted in the absence of a (CC's_GG) sequence recognized by the NciI restriction enzyme. Therefore, amplification of genomic DNA (primers were identical to those used for screening) with subsequent cleavage by NciI yielded 65 bp and 159 bp fragments for wild-type allele, 224 bp uncleaved product for the targeted allele (Fig 1E).

### PLZF protein expression determined by Western blotting

N-terminal rabbit monoclonal anti-PLZF antibody (ab189849) was purchased from Abcam (Cambridge, UK). Monoclonal mouse α-tubulin (B-5-2-1) was from Sigma-Aldrich (St. Louis, Missouri, USA). Membranes were incubated overnight at 4°C with antibodies at final dilution 1:5000 (PLZF) or 1:15000 (α-tubulin), secondary HRP-conjugated antibody was from GE Healthcare Bio-Sciences (Little Chalfont, UK), and signal was detected using ECL Prime chemiluminiscent detection kit (GE Healthcare Bio-Sciences) and Hyperfilm ECL. Developed hyperfilms were scanned and densitometry performed in ImageJ. We made 4 technical replicates and used normalized average of PLZF/control protein density as an estimate of expression level of PLZF in each rat. Theoretical molecular weight of unmodified PLZF is 74 kDa. We observed signal consistently at slightly more than 80 kDa, in skeletal muscle there can also be a genuine minor isoform of approximately 60 kDa (absent in null homozygotes). In liver there is a cross-reacting protein of approximately 100 kDa, which most probably does not represent a PLZF isoform, since there is no evidence in mammalian genomes/transcriptomes of such larger coding sequence.

### Prenatal study

The animals were mated overnight. The day, when a sperm was present in a vulva early morning was determined as the first day of pregnancy corresponding to the first day of embryonic development. We totally harvested 37 fetuses at embryonic day (ED) 20–21. After the harvesting, each fetus was weighed, and a sample of tissue was taken from its external ear for determination of its genotype. 9 *Plzf*^*-/-*^, 17 *Plzf*^*+/-*^, and 11 *Plzf*^*+/+*^ rats were investigated.

The native fetuses were examined under stereo-loupe (Leica Microsystems GmbH, Wetzlar, Germany) to detect external malformations. The gross malformations of the internal organs were evaluated after opening the thoracic and abdominal cavities. The organs exhibiting pathological changes were fixed in Bouin-Hollande fluid for 7–10 days, routinely histologically processed, and 8μm thick sections were stained by alcian blue-haematoxylin-eosin. For detection of skeletal anomalies, the specimens were fixed in graded series of ethanol (70–96%), and alcian blue-alizarin red skeletal staining was performed.

### Postnatal study

A 10-day-old *Plzf*^*-/-*^ rat and its 2 wild-type siblings were investigated. The external and internal examinations and further processing of the specimens were made in a similar way as in the prenatal study.

### Statistical analysis

Body weight data are expressed as means ± S.E.M. Differences between animals with *Plzf*^*-/-*^, *Plzf*^*+/-*^ and *Plzf*^*+/+*^ genotypes were analyzed by one away ANOVA with adjustments for multiple comparisons by Holm Sidak testing. Statistical significance was defined as P<0.05.
