# Correction: Targeting of the *Plzf* Gene in the Rat by Transcription Activator-Like Effector Nuclease Results in Caudal Regression Syndrome in Spontaneously Hypertensive Rats

**DOI:** 10.1371/journal.pone.0228139

**Published:** 2020-01-16

**Authors:** František Liška, Renata Peterková, Miroslav Peterka, Vladimír Landa, Václav Zídek, Petr Mlejnek, Jan Šilhavý, Miroslava Šimáková, Vladimír Křen, Colby G. Starker, Daniel F. Voytas, Zsuzsanna Izsvák, Michal Pravenec

The following information is missing from the Funding statement: Dr. Zsuzsanna Izsvák was funded by European Research Council-2011-ADG-TRANSPOSOstress– 294742.
